# Hard X-ray wavefront correction via refractive phase plates made by additive and subtractive fabrication techniques

**DOI:** 10.1107/S1600577520007900

**Published:** 2020-07-30

**Authors:** Frank Seiboth, Dennis Brückner, Maik Kahnt, Mikhail Lyubomirskiy, Felix Wittwer, Dmitry Dzhigaev, Tobias Ullsperger, Stefan Nolte, Frieder Koch, Christian David, Jan Garrevoet, Gerald Falkenberg, Christian G. Schroer

**Affiliations:** a Deutsches Elektronen-Synchrotron DESY, Notkestrasse 85, 22607 Hamburg, Germany; b Department Physik, Universität Hamburg, Luruper Chaussee 149, 22761 Hamburg, Germany; c Ruhr-Universität Bochum, Universitätsstrasse 150, 44801 Bochum, Germany; d Institute of Applied Physics, Abbe Center of Photonics, Friedrich-Schiller-Universität Jena, Albert-Einstein-Strasse 15, 07745 Jena, Germany; e Fraunhofer Institute for Applied Optics and Precision Engineering, Albert-Einstein-Strasse 7, 07745 Jena, Germany; f Laboratory for Micro- and Nanotechnology, Paul Scherrer Institute, 5232 Villigen PSI, Switzerland

**Keywords:** refractive X-ray optics, aberration correction, ptychography, phase plate

## Abstract

Modern subtractive and additive manufacturing techniques present new avenues for X-ray optics with complex shapes and patterns. Refractive phase plates acting as glasses for X-ray optics have been fabricated, and spherical aberration in refractive X-ray lenses made from beryllium has been successfully corrected. A diamond phase plate made by femtosecond laser ablation was found to improve the Strehl ratio of a lens stack with a numerical aperture (NA) of 0.88 × 10^−3^ at 8.2 keV from 0.1 to 0.7. A polymer phase plate made by additive printing achieved an increase in the Strehl ratio of a lens stack at 35 keV with NA of 0.18 × 10^−3^ from 0.15 to 0.89, demonstrating diffraction-limited nanofocusing at high X-ray energies.

## Abstract 2

Spherical aberrations in nanofocused X-ray beams generated by beryllium compound refractive lenses at 8.2 keV and 35 keV are corrected via refractive phase plates fabricated by subtractive and additive technology.

## 1. Introduction   

An increasing number of ultra-low-emittance storage-ring sources are coming into operation (Tavares *et al.*, 2018 ▸; Raimondi, 2016 ▸; Liu *et al.*, 2014 ▸) and more facility upgrades and new facilities are planned (Hettel, 2014 ▸; Streun *et al.*, 2018 ▸; Schroer *et al.*, 2018 ▸; Jiao *et al.*, 2018 ▸). The increase in brightness as a result of the strongly reduced source size and divergence will foster new science, if X-ray optics can preserve the beam properties. Due to higher lateral coherence, a larger portion of the X-ray beam can be used for diffraction-limited focusing. However, this requires optics with a sufficiently large aperture to collect the coherent beam. At the same time a high shape fidelity is required for diffraction-limited focusing. A similar challenge arises at X-ray free-electron lasers (XFELs), where ultrashort pulses and extremely high X-ray intensities are utilized, which puts the radiation resistance of current optics to the test (Uhlén *et al.*, 2013 ▸; Koyama *et al.*, 2016 ▸).

Parabolic compound refractive X-ray lenses (CRLs; Lengeler *et al.*, 1999 ▸) represent one possible solution for those problems, as they have a large aperture, high radiation resistance, and diffraction-limited focusing capabilities well below 100 nm. Over the past decades mechanically pressed beryllium (Be) lenses have been widely employed for beam transport and conditioning (Heimann *et al.*, 2016 ▸), but also for nanofocusing at XFEL sources (Schropp *et al.*, 2013 ▸; Seiboth *et al.*, 2014*b* ▸). Technical advances have enabled the microfabrication of diamond lenses by ion/plasma etching (Alianelli *et al.*, 2010 ▸; Lyubomirskiy *et al.*, 2019*a* ▸), laser ablation (Terentyev *et al.*, 2017 ▸; Antipov *et al.*, 2018 ▸), or focused ion beam milling (Medvedskaya *et al.*, 2020 ▸). Another new approach is the additive manufacturing of lenses by two-photon polymerization (Petrov *et al.*, 2017 ▸; Lyubomirskiy *et al.*, 2019*b* ▸). So far, all techniques struggle with inherent limitations of the fabrication process and the production of X-ray optics with diffraction-limited performance, high numerical aperture (NA), large geometrical aperture, and sufficient radiation resistance is non-trivial. However, a constant evolution of these techniques offers not only the capability to fabricate lenses but also the freedom to produce almost any shape. Thus, new refractive optical elements for aberration correction (Sawhney *et al.*, 2016 ▸; Seiboth *et al.*, 2017 ▸; Laundy *et al.*, 2019 ▸) and wavefront manipulation (Seiboth *et al.*, 2019 ▸) have emerged. They provide an alternative to deformable mirrors (Mimura *et al.*, 2010 ▸) and differential deposition methods (Matsuyama *et al.*, 2018 ▸) for wavefront correction as well as to diffractive elements (Vila-Comamala *et al.*, 2014 ▸; Loetgering *et al.*, 2020 ▸) for wavefront manipulation.

Here, we investigate two possibilities to fabricate glasses for X-ray optics: Femtosecond laser ablation in diamond and printing by two-photon absorption in a polymer. Compared with our previous work the transition from ablation in fused silica (Seiboth *et al.*, 2017 ▸) to diamond will enhance phase plate transmission and reduce the aspect ratio due to superior material properties of diamond. While polymer printing already showed excellent results in the past (Schropp *et al.*, 2018 ▸, 2019 ▸), the potential of this fabrication technique is further explored by printing phase plates with high aspect ratios that are required for aberration correction at high X-ray energies or to compensate for very strong wavefield deformation. Shape accuracy and surface properties of the fabricated phase plates are discussed, as well as their potential radiation resistance. The phase plates are then employed to correct spherical aberration of Be CRLs in two different nanofocusing scenarios. At a lower X-ray energy of 8.2 keV we demonstrate focusing of X-rays by a lens with a NA of 0.88 × 10^−3^. At a high X-ray energy of 35 keV we achieve sub-100 nm focusing by a lens with a NA of 0.18 × 10^−3^, giving a perspective for possible applications at fourth-generation sources with ultra-low emittance.

## 2. Experimental setup   

To measure the focusing properties of the X-ray optics, we use ptychography (Thibault *et al.*, 2008 ▸; Maiden & Rodenburg, 2009 ▸), a scanning coherent diffraction imaging method used routinely to retrieve the complex wavefield for optics characterization (Schropp *et al.*, 2010 ▸; Kewish *et al.*, 2010*a* ▸,*b* ▸; Vila-Comamala *et al.*, 2011 ▸; Hönig *et al.*, 2011 ▸; Schropp *et al.*, 2013 ▸; Uhlén *et al.*, 2014 ▸; Seiboth *et al.*, 2014*a* ▸; Kubec *et al.*, 2014 ▸; Morgan *et al.*, 2015 ▸; Patommel *et al.*, 2017 ▸). In particular, the performance of Be CRLs can be determined quantitatively. Ptychography works best if source size effects can be neglected, *i.e.* the X-ray beam illuminating the lens aperture is laterally coherent. In this case, the focus is dominated by diffraction. Its size and shape depends on the NA of the lens and any aberrations of the optics, rather than on the demagnified X-ray source size. Following Schroer & Falkenberg (2014 ▸), the regime of diffraction-limited focusing of a refractive lens is reached when the effective aperture *D*_eff_ of the optics is smaller than the lateral coherence length,
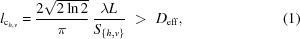
where λ is the X-ray wavelength, *L* is the source-to-optics distance and *S*_{*h*,*v*}_ is the full width at half-maximum (FWHM) source size in the horizontal and vertical direction, respectively. In the following, the coherence properties are estimated for the given experimental conditions.

The Be CRLs used in these experiments were manufactured by RXOPTICS from beryllium IF-1 foils (Materion) with 0.5 mm thickness. The radius of curvature for these bi-concave lenses measures 50 µm and their geometrical aperture *D* = 300 µm. The effective aperture *D*_eff_ is reduced compared with the geometrical aperture *D* by the attenuation inside the lens material (Lengeler *et al.*, 1999 ▸; Schroer & Falkenberg, 2014 ▸) and depends on the particular experimental conditions.

For the experiments at 8.2 keV a stack of *N* = 50 lenses was used. It has an effective aperture of *D*_eff_ = 191 µm. For the experiments at 35 keV the lens stack comprised *N* = 149 lenses with an effective aperture of *D*_eff_ = 236 µm.

### 2.1. Nanofocusing at 8.2 keV   

**Figure 1:**
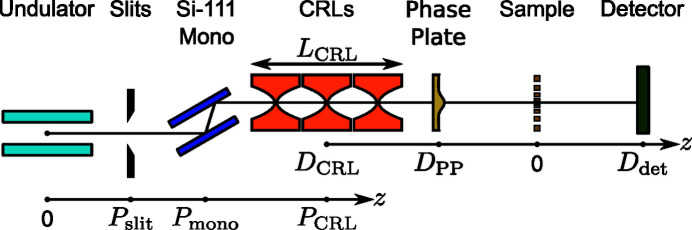
Schematic of the beamline setup at both I13-1 of DLS and P06 of PETRA III. At I13-1 we used a stack of 50 Be CRLs at 8.2 keV with a total length of *L*_CRL_ = 55 mm. At P06 we used 149 Be CRLs at 35 keV with a stack length of *L*_CRL_ = 298 mm. All other distances can be found in Table 1 ▸.

Experiments were carried out at the coherence branch I13-1 (Rau *et al.*, 2011 ▸) of Diamond Light Source (DLS) at a photon energy of 8.2 keV. A sketch of the beamline layout can be seen in Fig. 1 ▸ and relevant distances are listed in Table 1 ▸. The FWHM X-ray source size created by the undulator measures 400 µm × 13 µm (h × v). The vertical source is located in the center of the undulator; the horizontal source has its minimum size 11 m further downstream, which is 212 m upstream of the Be CRLs. According to equation (1) the lateral coherence length 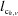 at the lens aperture is 60 µm × 1942 µm (h × v). In order to satisfy 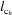 > 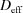 = 191 µm we closed horizontal slits at *P*_slit_ = 20 m downstream of the undulator to 30 µm, increasing the lateral coherence length at the Be CRLs to 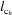 = 766 µm. X-rays were focused by the stack of 50 Be CRLs with a total length of *L*_CRL_ = 55 mm, resulting in a focal length of 108.6 mm and NA of 0.88 × 10^−3^. The difference in the horizontal and vertical source position leads to astigmatic focusing. In the given geometry, however, the focal positions for the vertical and horizontal directions differ by less than the depth of focus. The effect is thus negligible.

**Figure 2:**
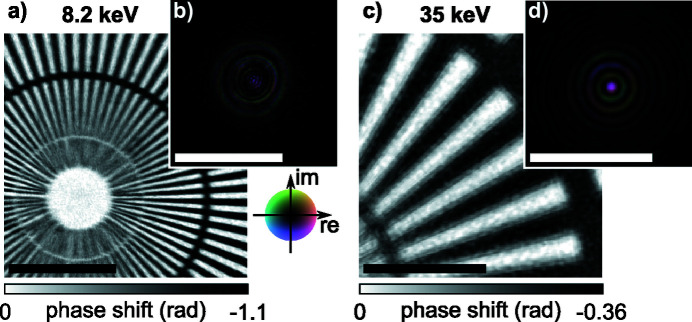
Ptychographic reconstructions of the test objects and illuminating wavefields obtained at 8.2 keV (*a*, *b*) and 35 keV (*c*, *d*). (*a*) Reconstructed object phase shift of a Siemens star patterned into a 500 nm-thick gold layer. (*b*) Reconstructed complex illumination function in the object plane. Both images in (*a*) and (*b*) are shown at the same scale and the bar represents 4 µm. (*c*) Reconstructed object phase shift of a Siemens star patterned into a 500 nm-thick tantalum layer. (*d*) Reconstructed complex illumination function in the object plane. Both images in (*c*) and (*d*) are shown at the same scale and the bar represents 2 µm.

The sample, a Siemens star pattern made out of 500 nm-thick gold on a 200 nm silicon nitride support with 50 nm smallest features, was placed roughly 1 mm out of focus. We scanned the sample with a step size of 1 µm across the beam, covering an area of 9 µm × 9 µm with 10 × 10 scan points. Far-field diffraction patterns with a dwell time of 1 s were recorded with a quad chip Merlin photon-counting pixel detector (Plackett *et al.*, 2013 ▸) at a distance of 3.675 m downstream of the sample. The detector images were cropped to 446 × 431 pixels and zero-padded to 512 × 512 pixels, so that the optical axis is centered. With a detector pixel size of 55 µm the resulting pixel size in the reconstructed images is 19.7 nm. An example of the reconstructed object phase shift and complex probe field at the object position is shown in Figs. 2 ▸(*a*) and 2(*b*), respectively. From the reconstructed object shown in Fig. 2 ▸(*a*) one can clearly see the impact of mechanical instabilities in the horizontal direction, leading to a degradation in contrast for the vertically oriented spokes of the Siemens star.

### 2.2. High-energy nanofocusing at 35 keV   

Measurements were carried out at the microprobe end­station of beamline P06 of PETRA III at DESY at a photon energy of 35 keV. A sketch of the beamline layout can be seen in Fig. 1 ▸ and relevant distances are listed in Table 1 ▸. Here, the FWHM X-ray source size measures 85 µm × 14 µm (h × v) and is located 93 m upstream of the Be CRLs. According to equation (1) the lateral coherence length 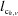 at the focusing optics measures 29 µm × 176 µm (h × v). To increase the horizontal coherence length we closed horizontal slits at *P*_slit_ = 27 m downstream of the undulator to 16 µm, yielding a lateral horizontal coherence length of 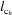 = 109 µm at the Be CRLs. Thus, at these high X-ray energies, the effective aperture *D*_eff_ = 236 µm was not illuminated coherently.

X-rays were focused by a stack of 149 Be CRLs with a total length of *L*_CRL_ = 298 mm, resulting in a focal length of 653.6 mm and NA of 0.18 × 10^−3^. A Siemens star test object made by NTT-AT (ATN/XRESO-50HC) with a 500 nm-thick tungsten layer and 50 nm smallest features was placed roughly 3 mm out of focus. The object was scanned with a step size of 200 nm and 400 nm, covering an area of 4 µm × 4 µm with 20 × 20 and 10 × 10 steps, respectively. An X-Spectrum Lambda 2M detector (Pennicard *et al.*, 2013 ▸) with a 500 µm GaAs sensor was used to record far-field diffraction patterns at a distance of 8.435 m downstream of the sample. With a cropping of 256 × 256 pixels around the optical axis and a detector pixel size of 55 µm, the pixel size in the reconstructed images equals 21.2 nm. An example of the reconstructed object phase shift and complex probe field at the object position is shown in Figs. 2(*c*) and 2(*d*) ▸, respectively. The reconstruction of the object is in part influenced by the reduced lateral coherence. However, main artifacts like the grainy background in Fig. 2 ▸(*c*) originate from the inhomogeneous response of the GaAs sensor.

### 2.3. GaAs sensor inhomogeneity   

**Figure 3:**
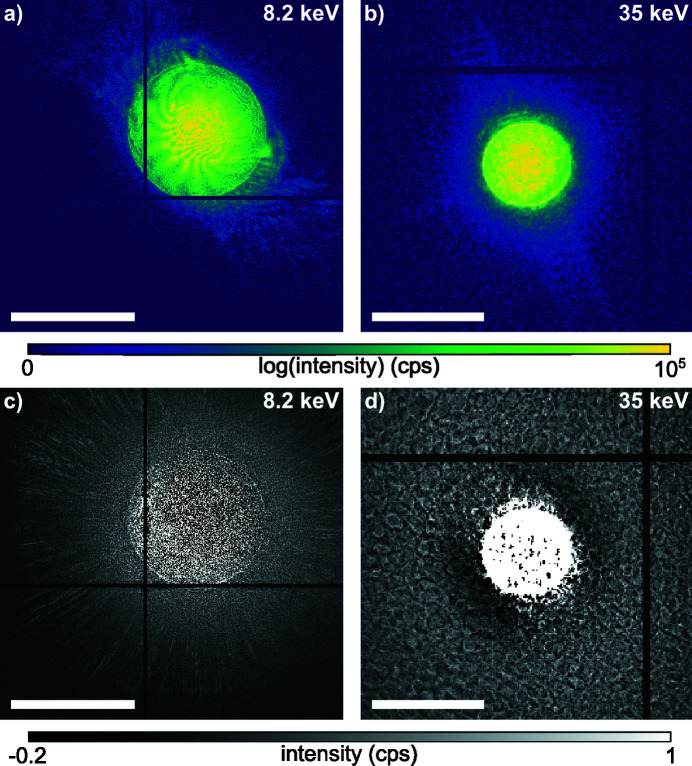
Example of diffraction patterns and average background signal, which is calculated from the difference between measured data and modeled diffraction patterns from ptychography. (*a*) Cropped diffraction pattern recorded with a Merlin detector at 8.2 keV. The beam is off-center as the detector ends in the upper right corner. (*b*) Cropped diffraction pattern at 35.0 keV recorded with the Lambda 2M GaAs detector. (*c*) Average background at 8.2 keV with the Merlin Si sensor. (*d*) Average background at 35 keV with the Lambda GaAs sensor. The scale bar in all images represents a scattering vector of 0.1 nm^−1^.

In Figs. 3 ▸(*a*) and 3(*b*) a typical scattering pattern for the Merlin as well as for the Lambda detector at 8.2 keV and 35.0 keV is shown, respectively. An average background image is calculated from the difference between measured diffraction patterns and those modeled based on the ptychographic reconstruction (Bernert *et al.*, 2017 ▸). The resulting background that corresponds to the reconstructions shown in Fig. 2 ▸ is shown in Figs. 3 ▸(*c*) and 3(*d*) for the Merlin detector at 8.2 keV and for the Lambda detector at 35.0 keV, respectively. While the Merlin background in Fig. 3 ▸(*c*) mainly shows incoherent scattering, the Lambda background in Fig. 3 ▸(*d*) shows a clear pattern originating from inhomogeneities in the GaAs sensor material. Although a flat-field correction was applied to the diffraction patterns, the large variations in the sensitivity of the sensor locally distort the diffraction patterns at low count rates. In addition to the reduced coherence at higher X-ray energies, these features of the sensor material hamper the convergence of the ptychographic algorithm, leading to stronger reconstruction artifacts in the object as shown in Fig. 2 ▸(*c*). As varying sensitivity effects are more dominant for lower count rates at high scattering angles, reconstruction artifacts are mainly at high spatial frequency. As the probe, shown in Fig. 2 ▸(*d*), is kept constant within the ptychographic algorithm across all scan positions (Maiden & Rodenburg, 2009 ▸), inconsistencies are pushed into the object and the probe wavefield is nevertheless reconstructed with confidence.

## 3. Refractive phase plates   

**Figure 4:**
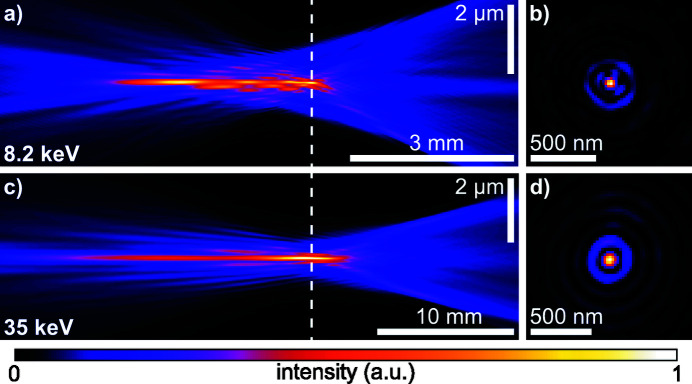
Characterized X-ray beams with spherical aberration. (*a*) Horizontal beam caustic at 8.2 keV. (*b*) Intensity distribution in the plane with highest peak intensity, marked by the dashed line in (*a*). (*c*) Horizontal beam caustic at 35 keV. (*d*) Intensity distribution in the plane with highest peak intensity, marked by the dashed line in (*c*).

From the initial wavefield characterization via ptychography, as shown in Fig. 2 ▸, one can numerically propagate the complex wavefield along the optical axis using the Fresnel–Kirchhoff diffraction integral. In Figs. 4 ▸(*a*) and 4(*c*) the beam caustic in the horizontal direction is shown for 8.2 keV and 35 keV, respectively. The caustic is created by projecting the intensity of the three-dimensional wavefield of the beam onto the horizontal plane. A dashed line in Fig. 4 ▸(*a*) and 4(*c*) marks the plane with the highest peak intensity, which was assumed to be the principal focal plane. It is shown in Figs. 4 ▸(*b*) and 4(*d*) for 8.2 keV and 35 keV, respectively. The beam caustics in Figs. 4 ▸(*a*) and 4(*c*) show areas further upstream with high intensity on the optical axis. These secondary focal planes originate from a varying curvature of the Be CRL along its rotational parabolic profile. More specifically, the inner part close to the optical axis of the Be CRL seems to be more strongly curved, leading to a shorter focal length for X-rays impinging close to the optical axis on the lens aperture (Schropp *et al.*, 2013 ▸). This spherical aberration does not lead to a broadening of the focal spot, but rather creates strong side lobes around the central speckle, as shown in Figs. 4 ▸(*b*) and 4(*d*). To correct for these aberrations, which are caused by peak-to-valley (PV) shape inaccuracies of roughly 0.5 µm in the printing tools used during lens manufacturing (Seiboth *et al.*, 2017 ▸), we pursued the concept of an additional optical element to correct for any lens aberrations. Here, we use a phase plate based on refraction (Seiboth *et al.*, 2017 ▸), similar to glasses for the correction of eyesight. An ideal focusing lens creates a converging spherical wave at its exit. Aberrations lead to a deformation of this ideal phase profile. The wavefield Ψ_Δ*z*_(*x*, *y*) at any plane behind the lens can be determined from the measured complex illumination function via ptychography [see Figs. 2 ▸(*b*) and 2(*d*)] and by backpropagation of a distance Δ*z* along the optical axis. Subtracting a spherical wave φ_*r*_(*x*, *y*) = −*k*[(*r* ^2^ + *x* ^2^ + *y* ^2^)^1/2^ − *r*] with radius *r* and wavenumber *k* = 2π/λ, which is fitted to the wavefront curvature, yields the residual wavefront error,
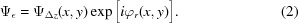
The thickness profile *z*_pp_(*x*, *y*) of the phase plate is designed to compensate any wavefront errors in Ψ_∊_ by introducing a phase shift φ_pp_ = −*k*δ_pp_(*k*)*z*_pp_(*x*, *y*) via refraction, where δ_pp_(*k*) is the refractive index decrement of the phase plate material, so that
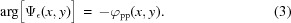
The calculated phase plate profile *z*_pp_(*x*, *y*) is only valid in the specific plane along the optical axis and at the measured photon energy. Since refractive power of the Be CRL changes with energy, the numerical aperture and convergence of the wavefield inside and after the Be CRL also changes. However, by translating the phase plate along the optical axis by a certain distance, one can compensate these effects and the phase plate can correct over a broad energy range (Seiboth *et al.*, 2018 ▸). Instead of measuring the wavefield for a specific lens combination, one can also pursue the approach to characterize the thickness profile of individual lens elements (Celestre *et al.*, 2020 ▸). This allows to numerically calculate any lens stack from the measured single lenses at arbitrary photon energies and to retrieve the potential wavefront deformation of this lens configuration numerically. Another approach for optical elements with very specific types of aberrations, like X-ray mirrors based on external total reflection, allows for the design of adaptable refractive elements to compensate the typical sinusoidal wavefront error found in these X-ray optics (Laundy *et al.*, 2019 ▸).

### 3.1. Diamond phase plates   

**Figure 5:**
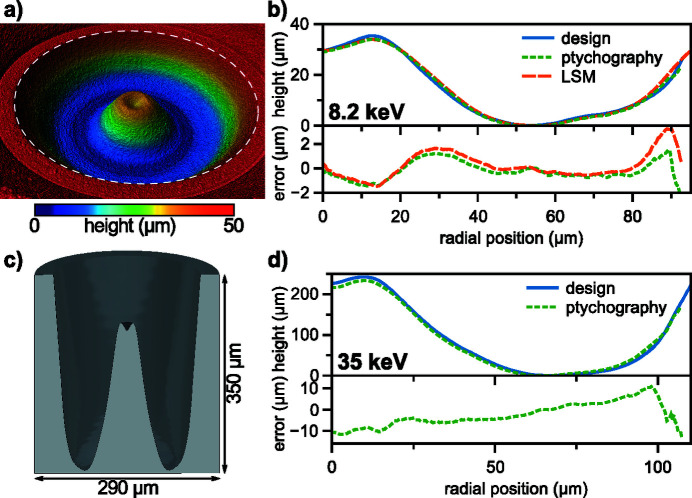
Refractive phase plates and their thickness profiles. (*a*) Surface of the diamond phase plate used at 8.2 keV, acquired by a Keyence VK-X1100 laser scanning microsope (LSM). The dashed circle represents a diameter of 220 µm. The optically relevant region of the phase plate has a smaller diameter of only 186 µm. (*b*) Radial height profiles of the diamond phase plate shown in (*a*) measured via ptychography (green, dotted line) and LSM (orange, dashed line) in comparison with the design goal (blue, solid line). The error for both measurements against the design goal is shown in the lower subplot. (*c*) 3D rendering of the polymer phase plate used at 35 keV. The model is sliced in the middle for better visibility of the characteristic phase plate shape. (*d*) Radial height profile of the polymer phase plate shown in (*c*) measured via ptychography (green, dotted line) in comparison with the design goal (blue, solid line). The error against the design goal is shown in the lower subplot.

**Figure 6:**
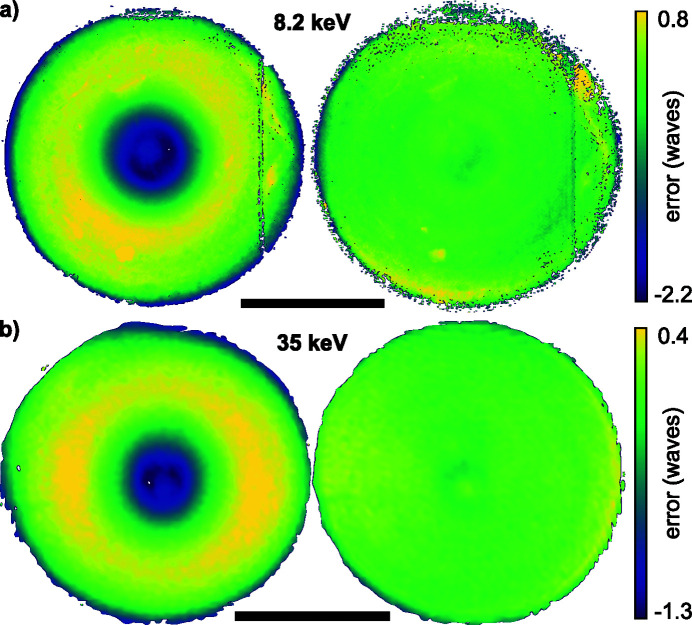
Residual wavefield error in the plane of the phase plate. (*a*) Wavefield error in the plane located 10.5 mm downstream of the lens exit aperture of 50 Be CRLs at 8.2 keV with (right) and without (left) corrective phase plate. (*b*) Wavefield error in the plane located 20 mm downstream of the lens exit aperture of 149 Be CRL at 35 keV with (right) and without (left) corrective phase plate. The scale bar in both figures represents 100 µm.

Diamond phase plates were manufactured from chemical vapour deposition (CVD)-grown single-crystal diamond substrates by Element Six measuring 2.6 mm × 2.6 mm × 0.3 mm with 〈100〉 orientation. For ablation, we used an Amplitude Satsuma fiber laser with second-harmonic generation module. Pulses with a duration of 300 fs and 515 nm wavelength were focused with a 20× objective (NA = 0.4) onto the substrate. To reduce absorption of the X-ray beam, the diamond substrate was thinned by 76 µm in a first step with a laser repetition rate of 12 kHz and pulse energy of 160 nJ. Therefore, layers with a thickness of 0.5 µm were sequentially ablated by scanning the focus across the sample surface with a hatch spacing of 0.5 µm by moving the substrate and the objective with a high-precision three-axis motion system from Aerotech (ANT130 XY, LZ) with a scanning velocity of 0.3 mm s^−1^. Subsequently, the phase-plate structure was stepwise ablated with a repetition rate of 6 kHz and pulse energy of 50 nJ, similar to the procedure described by Seiboth *et al.* (2017 ▸). For this, the profile was sliced into 70 layers and the substrate was ablated only at locations determined by the design. A surface profile of the manufactured diamond phase plate, acquired by a Keyence VK-X1100 laser-scanning microscope (LSM) with 50× objective and NA = 0.95, is shown in Fig. 5 ▸(*a*). The radially averaged profile from LSM measurements is compared with the design goal in Fig. 5 ▸(*b*). The height profile from ptychography shown in Fig. 5 ▸(*b*) is calculated from the difference of the backpropagated wavefields without phase plate [see Fig. 6 ▸(*a*), left side] and with phase plate [see Fig. 6 ▸(*a*), right side]. Measured profiles agree well with each other and closely match the design goal to within 2 µm, as shown in the lower subplot of the error against the design goal for both measurements in Fig. 5 ▸(*b*). Due to the ablation process the surface is typically not smooth. A surface roughness of *s*_a_ = 0.32 µm was determined by LSM measurements.

### 3.2. Polymer phase plates   

For the correction of 149 Be CRLs at 35 keV a peak wavefront error of 9.5 rad close to the optical axis had to be compensated. Due to very weak refraction at these high photon energies the aspect ratio of the resulting phase plate becomes critical for ablation techniques. Instead, we used additive manufacturing via two-photon polymerization in a Nanoscribe IP-S resist, as demonstrated by Schropp *et al.* (2018 ▸) and Seaberg *et al.* (2019 ▸). The structure was written with a Nanoscribe Photonic Professional GT in dip-in lithography mode with a 25× objective (NA = 0.8). Slicing and hatching of the three-dimensional structure was performed with a spacing of 200 nm. For fast lateral writing a galvo scanner system was used. Vertical displacement was achieved by a mechanical drive, as the piezo drive did not provide enough travel for the structure to be written without stitching. A three-dimensional model of the phase plate, which is sliced in the middle, is shown in Fig. 5 ▸(*c*). The outer ring with 350 µm height is printed for alignment purposes only and is not optically relevant for aberration correction. In Fig. 5 ▸(*d*) the relevant radial profile for aberration correction is shown. As the whole shape profile of the polymer phase plate could not be measured accurately by the LSM, only a height reconstruction from ptychography is shown. A thin Au coating of the polymer structure could potentially improve the reflectivity and thus the measurement success by LSM, but the steep slope angles in this particular case are an additional challenge. Instead, the profile is retrieved by subtracting the wavefield in the plane of the phase plate from two different ptychography measurements shown in Fig. 6 ▸(*b*), as described earlier in Section 3.1. The shape agrees well with the design, but the height scale does not fully match: the difference in PV height is about 10 µm [see the lower error plot in Fig. 5 ▸(*d*)] and corresponds to a <5% systematic error. There are two potential influencing factors for this systematic error: a deviation of the height scale of the printed structures relative to the nominal ones and a deviation of the index of refraction decrement δ_pp_ from the value used for the design. While the LSM could not follow the full profile on the steepest slopes it was possible to measure the flatter areas of the phase plate at the bottom and on the top of the central cone: at the radial position of 10 µm on the central cone a height of 246 µm was measured relative to the lowest points. This is to be compared with 242 µm of the design and with 232 µm calculated with the nominal δ_pp_ from ptychography. This indicates that δ_pp_ of the IP-S resist might be 2.124 × 10^−7^ at 35 keV instead of 2.216 × 10^−7^ used for the modeling. Since there are no precise measurements of the polymer’s refractive index in the X-ray regime, and both the density and mass proportions of the different photoresist constituents are not precisely known, an error in the estimated δ_pp_ is in fact expected.

## 4. Results   

Aberrated Be CRL stacks at both 8.2 keV and 35 keV were corrected by placing the respective phase plate directly behind the lens casing. As noted in Table 1 ▸ the phase plate was positioned at a distance of *D*_pp_ in front of the sample. Together with the position of the lens stack center *D*_CRL_ and the lens length *L*_CRL_ the phase plates were mounted |*D*_CRL_| − |*D*_Cpp_| − *L*_CRL_/2 = 10.5 mm and 20 mm after the lens exit for 8.2 keV and 35 keV, respectively. They were fixed along the optical axis and aligned perpendicular to the X-ray beam to within 2 µm. In a first step they were aligned to better than 5 µm by eye with the help of a scintillator-based high-resolution X-ray microscope in transmission geometry. Afterwards, wavefront characterization via ptychography was carried out and the position of the phase plate adjusted by typically 2 µm or less to reach best focus. Once aligned, the position was stable over several days and no further realignment was required.

### 4.1. Focusing at 8.2 keV   

**Figure 7:**
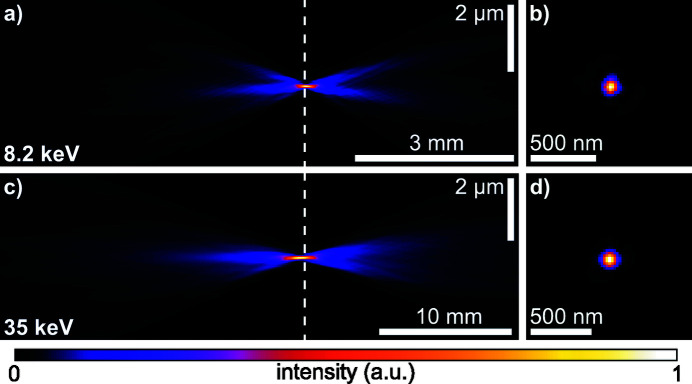
X-ray beams after correction with a phase plate as characterized by ptychography. (*a*) Horizontal beam caustic at 8.2 keV after correction with the diamond phase plate shown in Fig. 5 ▸(*a*). (*b*) Intensity distribution in the plane with highest peak intensity, marked by the dashed line in (*a*). (*c*) Horizontal beam caustic at 35 keV after correction with the polymer phase plate shown in Fig. 5 ▸(*c*). (*d*) Intensity distribution in the plane with highest peak intensity, marked by the dashed line in (*c*).

**Figure 8:**
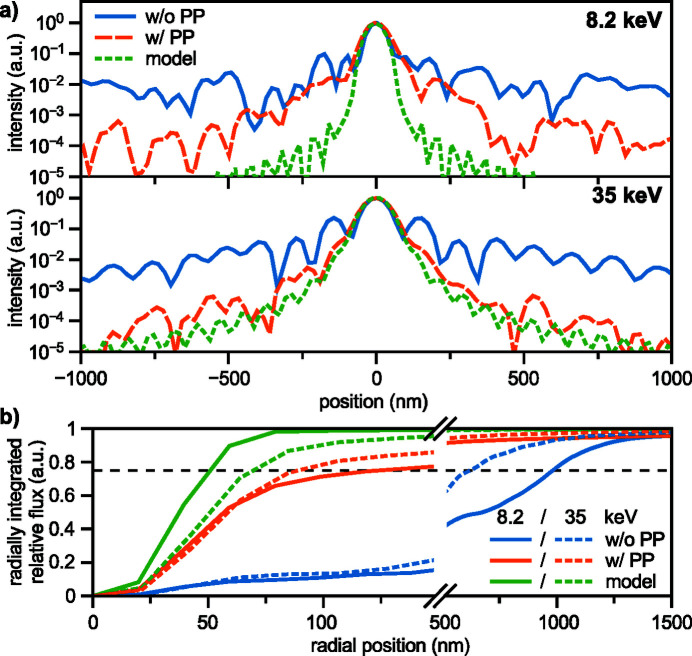
Intensity distribution in the focal plane. (*a*) Upper subplot: horizontal beam profile at 8.2 keV with the diamond phase plate (orange, dashed line) compared with the aberrated (blue, solid line) and ideal (green, dotted line) lens. Lower subplot: horizontal beam profile at 35 keV with the polymer phase plate (orange, dashed line) compared with the aberrated (blue, solid line) and ideal (green, dotted line) lens. (*b*) Radially integrated intensity distribution at both 8.2 keV (solid lines) and 35 keV (dotted lines), comparing the aberrated lens (blue) with the phase plate corrected (orange) and ideal (green) lens. The dashed horizontal line marks 0.75.

Before aberration correction, the wavefront error 10.5 mm downstream of the lens [left side in Fig. 6 ▸(*a*)] showed the typical rotational symmetry of spherical aberration with a PV error of 3.5 λ and root-mean-square (RMS) error of 0.75 λ. After alignment of the diamond phase plate shown in Figs. 5 ▸(*a*) and 5(*b*), the wavefront distortion reduced to a PV error of 1.11 λ and RMS error of 0.26 λ, shown on the right side in Fig. 6 ▸(*a*). Remaining errors are dominated by lens defects, visible as yellow spots in the lower part and on the right side of the wavefield in Fig. 6 ▸(*a*). The central area of the corrected wavefield indicates a slight shape mismatch of the phase plate compared with the design goal, as shown in Fig. 5 ▸(*b*). A vertical stripe on the right side of the lens aperture originates from the panel gap of the Merlin detector going through the direct beam in the diffraction patterns [see Fig. 3 ▸(*a*)]. The resulting beam caustic and focal spot of the corrected lens is shown in Figs. 7 ▸(*a*) and 7(*b*). X-rays are concentrated into a single focal plane marked by the dashed line, leading to a Gaussian focal spot with high intensity and reduced side lobes. The logarithmic plot in the upper part of Fig. 8 ▸(*a*) shows a suppression of side-lobe intensity between one and two orders of magnitude. The effect of side lobes and their impact on scanning microscopy techniques like fluorescence imaging becomes evident when comparing the radially integrated photon flux, shown in Fig. 8 ▸(*b*). Solid lines indicate the intensity distribution at 8.2 keV. While ∼75% of the intensity is homogeneously distributed over a radius of 986 nm for the aberrated lens (blue line), an improvement down to 138 nm with the phase plate (orange line) is observed. The theoretical value (green line) is at 52 nm. The FWHM focal spot size changed from 69 nm without the phase plate to 76 nm with the diamond phase plate. In the aberrated case the focal length varies with radius over the lens aperture. The convergent rays from outer parts of the lens aperture are focused into the plane marked by the dashed line in Fig. 4 ▸(*a*). Rays passing the lens close to the optical axis are focused further upstream. Thus, only a ring-shaped outer part of the lens contributes to the focus in the dashed plane. This aperture shape influences the width of the central speckle. As only large angles or high spatial frequencies contribute, the central speckle size is decreased (Kiss, 2016 ▸), but the strength of side lobes is enhanced at the same time due to missing lower spatial frequencies. If the whole lens aperture contributes, as is the case for the aberration-corrected lens in Fig. 7 ▸(*a*), the size of the central speckle increases slightly, but side lobes are suppressed. The Strehl ratio, measured as integrated intensity in the central speckle, increased from 0.10 to 0.70. As the diamond phase plate has a transmission of 75%, the total transmission of the optical system changed from 16.5% down to 12.4%. Focal spot parameters are also summarized in Table 2 ▸.

### 4.2. High-energy nanofocusing   

The large lens stack at 35 keV also shows the typical pattern of spherical aberration in a plane located 20 mm behind the lens exit [left side in Fig. 6 ▸(*b*)], but appears more homogeneous with no visible lens defects or dirt. The PV wavefront error is 1.7 λ and the RMS error 0.38 λ. After aligning the polymer phase plate [see Fig. 5 ▸(*c*)], the PV wavefront error reduced to 0.7 λ and the RMS value to 0.11 λ. In the central part of the aperture the slight height deviation of the fabricated phase plate [see Fig. 5 ▸(*d*)] is visible by the residual donut-shaped phase ring. A weak astigmatism, originating from the different source position in the horizontal and vertical direction due to the horizontal slits (see Fig. 1 ▸ and Table 1 ▸), can be seen by the slightly larger phase error in horizontal than in the vertical direction. The horizontal beam caustic after aberration correction is shown in Fig. 7 ▸(*c*). The dashed line marks the focal plane. Due to a slight astigmatism this plane falls between the best focus in the horizontal and vertical direction (separated by roughly 600 µm). Since the depth of focus is 700 µm, the influence on the focal spot size is small. A logarithmic plot of the focal spot profile in the horizontal direction is shown in the lower part of Fig. 8 ▸(*a*). Side lobes are suppressed by two orders of magnitude for the phase-plate-corrected lens (dashed, orange line) compared with the aberrated lens (blue, solid line) and follow closely the theoretical limit (green, dashed line). The influence of the radially integrated flux in focus is depicted in Fig. 8 ▸(*b*) by the dashed lines for 35 keV. Without a phase plate ∼75% of the radiation is spread over a radius of 637 nm. The phase plate improves this value to 90 nm (orange, dashed line) compared with the theoretical limit of 78 nm (green, dashed line). An increase in FWHM spot size is observed from 85 nm to 95 nm, mainly caused by astigmatism. The Strehl ratio improved from 0.15 to 0.89, indicating a diffraction-limited performance. Since the polymer is highly transparent for X-rays (transmission > 99%), the total lens transmission stays almost unchanged at 35%. A summary of the values can be found in Table 2 ▸.

**Figure 9:**
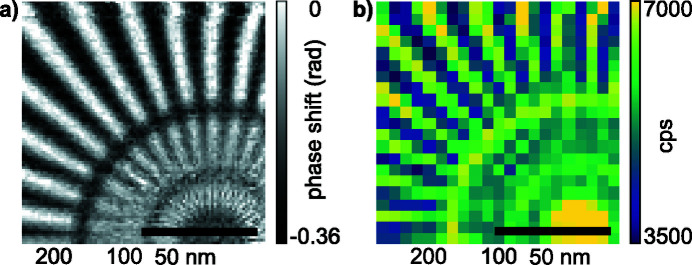
Resolution in scanning X-ray microscopy at 35 keV with corrective phase plate. (*a*) Reconstructed object phase shift via ptychography from the central part of a Siemens star test object. Spoke features range from 50 nm size in the innermost circle to about 200 nm for the outermost circle. (*b*) The same area as in (*a*), but in fluorescence contrast. The scan was performed with 100 nm steps with 21 × 21 scan points. The scale bar in both images represents 2 µm. The innermost spoke size in each radial segment is noted below the figures.

At high X-ray energies the lateral coherence of the storage-ring source decreases and diffraction-limited operation of large-aperture optics is challenging, as discussed in Section 2.2. Using spatial filtering, that is closing horizontal slits to 16 µm, the lateral coherence length is increased to enable ptychographic imaging. In this way, the shape of a coherent mode can still be retrieved from a wavefield reconstruction via ptychography [see Fig. 2 ▸(*b*)]. The real spot size is typically larger due to source effects. In Fig. 9 ▸(*a*) the reconstructed object of a Siemens star test sample (NTT-AT ATN/XRESO-50HC, see Section 2.2) is compared with the fluorescence signal in Fig. 9 ▸(*b*). The data were acquired during the same measurement, collecting far-field diffraction patterns and fluorescence signal at once. The resolution of Fig. 9 ▸(*a*) depends on the scattering angle in the diffraction patterns and is independent of beam size. Although the reconstruction suffers from a reduced lateral coherence, sample instability, and detector inhomogeneity [see Fig. 3 ▸(*d*)], the image provides a reference. The 100 nm-sized spokes in the middle ring are clearly resolved, whereas the 50 nm features in the innermost ring are not resolved due to data quality. The fluorescence map shown in Fig. 9 ▸(*b*) gives a good measure for the incoherent beam size, including source-size effects. The image was acquired in 21 × 21 steps with 100 nm step size. 200 nm-sized outermost spokes are clearly resolved. A focal spot size of about 260 nm FWHM was determined from fluorescence knife-edges on a different area of the same sample.

## 5. Conclusions   

We have demonstrated aberration correction of X-ray lenses via refractive phase plates made from two different materials and with complementary manufacturing techniques. In addition, aberration correction was successfully demonstrated not only for commonly used X-ray energies around 10 keV but also for high-energy X-rays at 35 keV. A stack of 50 Be CRLs with a NA of 0.88 × 10^−3^ at 8.2 keV was corrected by a single-crystal CVD-diamond phase plate fabricated via laser ablation. The Strehl ratio improved from 0.10 to 0.70, while the total transmission of the optical system decreased by 25% due to absorption within the phase plate. The achieved aberration-corrected focal spot size measures 76 nm FWHM. At 35 keV we corrected a stack of 149 Be CRLs with a NA of 0.18 × 10^−3^ by using a polymer phase plate manufactured additively via two-photon polymerization. The Strehl ratio improved from 0.15 to 0.89, indicating a diffraction-limited performance. The coherent focal spot size, neglecting source effects, measures 95 nm FWHM. Fluorescence knife-edge scans indicate a focal spot size of 260 nm, including source size effects.

Phase plates have become an important instrument to achieve better performance with refractive X-ray lenses at both storage-ring sources and XFELs. The two manufacturing approaches described here lead to complementary properties of optics. Laser ablation of diamond provides optics with a decent shape accuracy down to 1 µm, but the ablation process results in a large surface roughness. Subsequent etching or polishing (Antipov *et al.*, 2018 ▸) might be suitable to improve surface roughness in the future. The advantage of diamond is its high thermal conductivity in combination with low absorption, which makes these optics a promising choice for high-intensity applications at XFELs. Recent developments in the fabrication of diamond micro-CRLs using ion beam lithography (Medvedskaya *et al.*, 2020 ▸) could be a viable method to improve both shape accuracy and surface roughness for diamond optics.

Additive printing technology provides a high level of shape accuracy below 200 nm and very smooth surfaces. These optics are especially suited for storage-ring applications, where an operation over several weeks at beamline P06 showed a good radiation resistance. In addition, the material is highly transparent for X-rays. Whether the polymer phase plates can withstand several days of beam time at an XFEL remains an open question and might also strongly depend on the pulse train structure. At high X-ray energies above 20 keV the spot size is often limited by the typical source properties of third-generation storage-ring facilities when working with large-aperture optics. Fourth-generation ultra-low-emittance storage ring sources and hard XFELs that operate well above 20 keV in combination with aberration-corrected large-aperture optics will allow to collect a large fraction of the emitted light while efficiently focusing X-rays down to 100 nm and below, providing new opportunities for the investigation of thick samples and fluorescence imaging.

## Figures and Tables

**Table 1:** Position of relevant experimental components along the beamlines I13-1 of DLS (8.2 keV) and P06 of PETRA III (35 keV); the corresponding sketch of the setup can be seen in Fig. 1 ▸

		Position (m)†		Distance (m)‡	
Component	Symbol	I13-1	P06	I13-1	P06
Horizontal slits	*P*_slit_	20	27		
Monochromator	*P*_mono_	210	38		
Be CRL stack	*P*_CRL_ / *D*_CRL_	223	93	−0.107	−0.652
Phase plate	*D*_Cpp_			−0.069	−0.483
Detector	*D*_det_			3.675	8.435

† Position along beamline from undulator source.

‡ Relative distance to focal plane (*cf*. Fig. 1 ▸).

**Table 2:** Achieved focal spot size and Strehl ratio for the Be CRL and phase plate combinations investigated

Beamline	Energy (keV)	Phase plate	Focus size FWHM (nm)	Strehl ratio†	Transmission
DLS I13-1	8.2	–	69	0.10	0.165
DLS I13-1	8.2	Diamond‡	76	0.70	0.124
PETRA III P06	35.0	–	85	0.15	0.356
PETRA III P06	35.0	Polymer§	95	0.89	0.352

† Ratio of integrated intensity in central speckle compared to ideal, modeled lens.

‡ See Figs. 5(*a*) and 5(*b*) ▸.

§ See Figs. 5(*c*) and 5(*d*) ▸.
